# New Perspectives and Open Issues in the Adjuvant and Neoadjuvant Treatment of Melanoma

**DOI:** 10.3390/cancers18101669

**Published:** 2026-05-21

**Authors:** Andrea Spagnoletti, Lorenza Di Guardo, Alice Indini, Massimo Di Nicola, Roberto Patuzzo, Andrea Maurichi, Paolo Fava, Gabriele Roccuzzo, Alessandro Minisini, Federico Pravisano, Jacopo Pigozzo, Luisa Piccin, Carolina Cimminiello, Nikolaos Papadopoulos, Michele Del Vecchio

**Affiliations:** 1 Unit of Melanoma Medical Oncology, Department of Medical Oncology, Fondazione IRCCS Istituto Nazionale dei Tumori, 20133 Milan, Italy; andrea.spagnoletti@istitutotumori.mi.it (A.S.); lorenza.diguardo@istitutotumori.mi.it (L.D.G.); alice.indini@istitutotumori.mi.it (A.I.); 2 Immunotherapy and Innovative Therapeutics Unit, Department of Medical Oncology, Fondazione IRCCS Istituto Nazionale dei Tumori, 20133 Milan, Italy; massimo.dinicola@istitutotumori.mi.it; 3 Department of Melanoma Surgery, Fondazione IRCCS Istituto Nazionale dei Tumori di, 20133 Milan, Italy; roberto.patuzzo@istitutotumori.mi.it (R.P.); andrea.maurichi@istitutotumori.mi.it (A.M.); 4 Department of Medical Sciences, Section of Dermatology, University of Turin, 10124 Turin, Italy; paolo.fava@unito.it (P.F.); gabriele.roccuzzo@unito.it (G.R.); 5 Department of Medical Oncology, Santa Maria della Misericordia Academic Hospital, Azienda Sanitaria Universitaria Friuli Centrale (ASU FC), 33100 Udine, Italy; alessandro.minisini@asufc.sanita.fvg.it (A.M.); federico.pravisano@asufc.sanita.fvg.it (F.P.); 6 Medical Oncology 2, Veneto Institute of Oncology IOV-IRCCS, 35128 Padua, Italy; jacopo.pigozzo@iov.veneto.it (J.P.); luisa.piccin@iov.veneto.it (L.P.); 7 Melanoma Unit, Division of Clinical and Experimental Oncology and Immunotherapy of Melanoma, European Institute of Oncology, 20141 Milan, Italy; carolina.cimminiello@ieo.it (C.C.); nikolaos.papadopoulos@ieo.it (N.P.)

**Keywords:** melanoma, adjuvant therapy, neoadjuvant therapy, immunotherapy, immune checkpoint inhibitors, mRNA vaccine, biomarkers, gene expression profiling, circulating tumor DNA, recurrence risk

## Abstract

Melanoma adjuvant therapy has substantially improved recurrence-free and distant metastasis-free survival in patients with resected high-risk disease, and more recently, these advances have extended to earlier stages. However, important unmet needs remain, including the management of stage IIIA disease, the optimal treatment strategy after relapse on adjuvant therapy, and the identification of biomarkers capable of refining patient selection. This review summarizes recent advances and unresolved questions in the adjuvant and neoadjuvant treatment of melanoma. We discuss novel systemic strategies, including immune checkpoint inhibitor combinations and personalized neoantigen mRNA vaccines, together with the expanding role of neoadjuvant approaches. We also examine prognostic and predictive tools—such as clinicopathologic models, circulating tumor DNA, serum biomarkers, tumor microenvironment features, and gene expression profiling—that may help better define recurrence risk and therapeutic benefit. Current evidence suggests that although modern therapies have changed the natural history of resected melanoma, a substantial proportion of patients are still overtreated or undertreated when treatment decisions are based on stage alone. Future progress will depend on integrating biological risk stratification with clinical staging and optimizing treatment sequencing across adjuvant and neoadjuvant settings.

## Simple Summary

Melanoma treatment has evolved rapidly in recent years. Adjuvant anti-PD-1 therapy and, for BRAF-mutant disease, targeted therapy have improved recurrence-free outcomes in resected stage III and IV melanoma, and anti-PD-1 therapy has also proven effective in stage IIB–C disease. At the same time, several important questions remain unresolved, including whether and when to treat stage IIIA disease, the interpretation of relapse after adjuvant therapy, and the identification of patients most likely to benefit from treatment while sparing others unnecessary toxicity. This review discusses emerging strategies in both the adjuvant and neoadjuvant settings, including novel immunotherapy combinations, individualized neoantigen treatment (mRNA vaccines), and biomarker-driven risk stratification. It also highlights ongoing controversies related to surrogate endpoints, treatment sequencing, and the integration of molecular tools into clinical decision making. Overall, the field is moving toward increasingly personalized treatment approaches, although further evidence is required before many of these strategies can be routinely implemented.

## 1. Introduction

Adjuvant therapy (ADJ) has significantly improved recurrence-free survival (RFS) and distant metastasis-free survival (DMFS) in resected melanoma and has likely contributed to the recent decline in melanoma-related mortality. More recently, neoadjuvant immunotherapy (NIT) has emerged as a promising strategy for resectable stage IIIB/IV melanoma, with the potential to improve outcomes and enable response-adapted treatment. As treatment options expand, several uncertainties have emerged, including the management of stage IIIA disease, the most appropriate endpoints for evaluating benefit, the interpretation of pathologic response after NIT, and the optimal management of patients who relapse after ADJ/NIT treatment. Despite these advances, a substantial proportion of patients do not derive benefit from systemic therapy. Novel immunotherapy combinations, including mRNA-based cancer vaccines, are under investigation, and the development of predictive biomarkers may improve patient selection and optimize treatment across both the ADJ/NIT settings.

## 2. New Combinations: Promise and Disappointment in the Adjuvant Setting

### 2.1. ICIs Combinations

The success of combined immune checkpoint inhibitors (ICIs) in advanced cutaneous melanoma [1] provided a strong rationale for testing ICI combinations in the adjuvant setting. However, the results available so far have been largely disappointing. In the phase III CheckMate 915 trial, adjuvant nivolumab plus low-dose ipilimumab did not improve RFS compared with nivolumab alone in patients with resected stage IIIB-D or IV melanoma (24-month RFS 64.6% vs. 63.2%; HR 0.92), and no benefit was observed in the PD-L1 < 1% subgroup. Toxicity was substantially higher with the combination, with grade 3–4 treatment-related adverse events in 32.6% versus 12.8% of patients and treatment discontinuation in 31.6% vs. 10.4% [2]. One important exception is the phase II IMMUNED trial, conducted in a highly selected population of patients with resected stage IV melanoma with no evidence of disease. In that study, standard-dose nivolumab plus ipilimumab significantly improved RFS compared with placebo (HR 0.25; 4-year RFS 64.2% vs. 15.0%) and was also associated with an overall survival (OS) advantage versus placebo. Nevertheless, this benefit came at the cost of marked toxicity, with grade 3–4 treatment-related adverse events in 71% of patients. In addition, the trial was relatively small and allowed crossover, which limits the generalizability of its results to the broader adjuvant population [3]. More recent studies have reinforced this negative picture. In the phase III RELATIVITY-098 trial, adjuvant nivolumab plus relatlimab failed to improve RFS/DMFS over nivolumab alone after complete resection of stage III/IV melanoma (HR 1.01; 95% CI 0.83–1.22; *p* = 0.928). Toxicity was again higher with the combination, with grade 3/4 treatment-related adverse events in 19% versus 8% of patients and treatment discontinuation in 17% versus 9% of patients. Notably, translational analyses suggested that the absence of macroscopic tumor and the lower frequency of circulating LAG-3-positive T cells in the adjuvant setting may partly explain why a regimen active in metastatic melanoma did not provide additional benefit after complete resection [4]. A similar outcome was observed with TIGIT blockade. In the phase III KEYVIBE-010 trial, adjuvant vibostolimab plus pembrolizumab in resected high-risk stage IIB-IV melanoma crossed the prespecified futility boundary at the first interim analysis and was discontinued. The combination did not improve outcomes over pembrolizumab alone and was numerically inferior for RFS (HR 1.25; 6-month RFS 80% vs. 85%), while also causing more grade ≥3 treatment-related adverse events, more serious adverse events, and more treatment discontinuations [5]. Therefore, as of March 2026, no ICI combinations have demonstrated superiority over single-agent anti-PD-1 therapy in an unselected adjuvant melanoma population. Combination strategies remain investigational, including the phase III study of fianlimab plus cemiplimab versus pembrolizumab (NCT05352672), but anti-PD-1 monotherapy remains the adjuvant benchmark.

### 2.2. Cancer Vaccines: From Concept to Clinic

**Figure 1:**
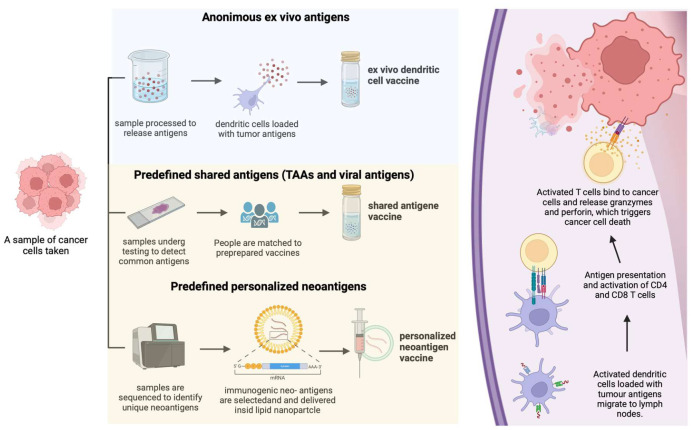
Main categories of cancer vaccines and proposed mechanism of action. Vaccines may use anonymous ex vivo antigens, predefined shared tumor-associated antigens, or patient-specific neoantigens. Personalized neoantigen approaches aim to prime and expand tumor-reactive T cells and their role in melanoma treatment is currently under study.

Vaccination is one of the oldest approaches developed to modulate the immune system, with the earliest attempts dating back more than a century [6,7,8]. Cancer vaccines can potentially target a broad range of intracellular antigens and prime tumor-reactive T cells. This property enables them to expand the spectrum of tumors that may benefit from immunological therapies, in contrast to ICIs, CAR-T cells, and bispecific T-cell engagers (BiTEs), which rely primarily on surface antigen repertoire and pre-existing T-cell infiltration. However, despite encouraging early-phase results, many cancer vaccines have failed to demonstrate clinical benefit in phase III randomized controlled trials. For instance, a vaccine targeting the common melanoma tumor antigen MAGE-A3 showed promising phase II activity, but did not confer a benefit over placebo in a subsequent adjuvant phase III trial [7,8]. Moreover, the rapid and dominant development of ICIs has contributed to a relative slowdown in cancer vaccine research. For example, treatment with the glycoprotein 100 (gp100) peptide vaccine with IL-2 improved response rate and PFS compared with IL-2 alone [9]. However, ipilimumab received regulatory approval in the same year based on a significant OS benefit, which was not enhanced by the addition of the gp100 vaccine. Consequently, further development of this vaccine strategy was discontinued. Several different types of antigens have been tested in clinical trials and can be broadly categorized into three groups: anonymous ex vivo antigens (e.g., dendritic cell-derived antigens); predefined shared antigens (including tumor-associated antigens, TAAs, and viral antigens); and predefined personalized neoantigens (Figure 1). Shared antigens are expressed in a sufficiently large proportion of patients to allow the development of “off-the-shelf” vaccines applicable to broad patient populations. Unfortunately, many trials evaluating vaccines based on shared antigens have failed to demonstrate meaningful clinical benefit. In contrast, the recent development of personalized neoantigen vaccines has the potential to overcome several limitations inherent to shared antigen approaches [10]. Personalized antigens are unique to each vaccinated patient, thereby overcoming the heterogeneous distribution of shared antigens. Moreover, targeting personalized antigens allows for exquisite specificity and activates T cells that bypass thymic negative selection, potentially mounting widespread T-cell reactivity in responding patients and promoting epitope spreading to new neoantigens [10,11]. Rapid advances in this field—driven by the success of mRNA vaccines (e.g., mRNA-1273) for SARS-CoV-2 and by the refinement and widespread adoption of next-generation sequencing (NGS) technologies—have enabled the translation of this promising strategy into the field of oncology. The breakthrough was achieved with the publication of the results of the first phase II study in the field, KEYNOTE-942. This randomized, open-label, phase II trial assessed a personalized mRNA-based cancer vaccine in combination with pembrolizumab in patients with resected stage IIIB-IV melanoma. The mRNA-based vaccine (mRNA-4157/V940), which encodes up to 34 patient-specific tumor neoantigens, is administered intramuscularly every three weeks. At 18 months, RFS was 78% in the combination arm compared with 62% in the single-agent pembrolizumab arm [HR = 0.561 (95% CI 0.309–1.017), *p* = 0.0266]. DMFS was also significantly improved, with rates of 91.8% in the mRNA vaccine group versus 76.8% in the pembrolizumab group [HR = 0.347 (95% CI 0.145–0.828), *p* = 0.0063] [12]. Although these data are promising, the RFS confidence interval approached/crossed unity, and the results should therefore be interpreted cautiously, particularly given the phase II design, the open-label nature of the study, and the relatively limited number of events. Updated 3-year follow-up presented at the ASCO 2024 suggested durable separation of the curves, with a 49% reduction in the risk of recurrence or death and a 62% reduction in the risk of distant metastasis or death, as well as an emerging trend toward improved OS (2.5-y OS 96.0% vs. 90.2%, HR 0.425) [12]. Importantly, immune-related adverse events appeared broadly comparable between treatment arms. Nevertheless, these findings remain hypothesis-generating until confirmed in a phase III trial. The data coming from the phase III study (NCT05933577) will be critical to determine the real favorable contribution of the addition of the mRNA-4157/V940 to pembrolizumab. Additional challenges include optimizing neoantigen selection, improving prediction of MHC binding and antigen presentation, shortening manufacturing timelines, reducing costs, and developing scalable infrastructures. Addressing these issues will be essential before personalized cancer vaccines can be widely implemented.

## 3. Open Issues in Neoadjuvant Therapy

NIT has increasingly emerged as a transformative approach for managing resectable stage IIIB-IV melanoma, offering more effective treatment options compared to traditional ADJ [13]. In the past, ADJ extended systemic treatment into earlier melanoma stages to eradicate microscopic residual disease after surgery. Although this approach has reduced recurrence risk, it treats patients uniformly without considering the individual biological and immunological characteristics of each patient’s tumor. Furthermore, adjuvant immunotherapy offers significant benefits in terms of RFS and DMFS, but uncertainties regarding OS benefits remain. Trials such as Keynote-054, which are expected to provide crucial OS information, have delayed their final analyses until 2027. Additionally, real-world studies have raised questions about the correlation between RFS/DMFS improvements observed in clinical trials and OS outcomes in routine clinical practice. Then, blocking the PD1/PDL1 pathway leads to the expansion of tumor-specific T-cell clones within the microenvironment of the tumor that is still present in situ and reverses the functional anergy of tumor-specific T cells in the tumor-draining lymph nodes [14]. This strategy has led to significant clinical advances, with several randomized trials validating its efficacy. For example, the SWOG S1801 study demonstrated that perioperative pembrolizumab significantly improved event-free survival (EFS) compared to adjuvant-only therapy [15]. Similarly, the NADINA trial showed superior EFS with low-dose neoadjuvant nivolumab plus ipilimumab, followed by response-adapted adjuvant treatment, compared to upfront surgery and adjuvant therapy alone [15]. Despite small differences in patient populations across these trials, such as the inclusion of <10% resectable stage IV melanoma patients in SWOG S1801, these data support NIT as a rapidly evolving and evidence-based option for appropriately selected patients with resectable macroscopic stage III/IV melanoma. However, NIT needs to be universally accepted as a standard of care. Despite this, several unresolved questions remain. One primary issue concerns the therapeutic strategy, nivolumab + ipilimumab, compared to anti-PD-1 monotherapy, which has shown higher 1-year EFS (84% vs. 72%) and pathological complete response (pCR) rates (48% vs. 38%). However, this comes with increased toxicity (29.7% vs. 12% for grade ≥ 3 adverse events). Another important issue is whether to use monotherapy vs. combination IO. The goal of future trials will consist of prospectively identifying and validating reliable biomarkers that allow us to characterize the TME as cold or hot in order to determine whether a less intensive or more aggressive treatment is warranted. Unlike metastatic disease, standard markers like BRAF status and PD-L1 expression have not proven predictive for the success of combo- versus mono-IO in the neoadjuvant setting. Current treatment decisions in NIT rely on subjective clinical parameters (e.g., tumor burden, lymph node involvement, and patient age) because reliable biological markers to guide therapy are lacking. Some exploratory analyses have linked molecular signatures such as IFN-γ and tumor mutational burden (TMB) to NIT outcomes. In the phase 2 PRADO trial, a selected biomarker-defined subgroup (high TMB, INF-γ and PD-L1) showed 100% major pathological response (MPR) and excellent long-term EFS [16]. In contrast, low expression of these markers was associated with poorer outcomes. However, these findings apply to exploratory subgroups rather than the full cohort and should be considered hypothesis-generating until prospectively validated. Future trials should focus on developing prospective, biomarker-driven studies to identify patient subgroups for tailored NIT approaches. A second important question is whether a risk-adapted strategy, as demonstrated in the NADINA and PRADO trials, should be adopted moving forward. Evidence from trials including NADINA, PRADO, and OPACINneo suggests that patients achieving MPR after neoadjuvant nivolumab plus ipilimumab may not need adjuvant therapy, given their excellent RFS and distant DMFS outcomes [15,17,18]. However, further validation is required, particularly for patients bearing either multiple positive nodes at baseline or higher lymph node tumor burden, as recurrences have been observed in this group despite achieving MPR. In fact, in the most recent NADINA trial update, patients achieving a near pCR had worse RFS at 2 years after surgery compared with those achieving a pPR (69.4% vs. 81.2%) [19]. However, these data should be interpreted with caution, and we need additional information and further characterization to define which patients can safely avoid adjuvant therapy. Third, the management of pathological partial responders and non-responders remains uncertain. In PRADO, patients with pPR had worse long-term EFS and DMFS than patients with pNR: a counterintuitive finding that likely reflects the response-adapted trial design. pPR patients generally underwent TLND alone, whereas pNR patients received TLND plus adjuvant systemic therapy, including BRAF/MEK inhibition when appropriate. The NADINA protocol was subsequently adapted to administer adjuvant therapy to patients with pPR, but further follow-up is needed to clarify the role and intensity of postoperative treatment in these heterogeneous subgroups.

Imaging techniques such as FDG-PET and tumor-derived biomarkers such as ctDNA may improve response prediction and risk stratification in future NIT algorithms. Beyond ICIs, localized neoadjuvant approaches are being investigated. Oncolytic viruses such as talimogene laherparepvec (T-VEC) have shown some efficacy and preliminary immune activity in early-phase studies, particularly when combined with PD-1 blockade, but their role remains investigational [20]. Additionally, intralesional immunocytokine therapy with Daromun (a combination of L19IL2 and L19TNF) has demonstrated favorable results in the PIVOTAL trial, showing significant improvements in RFS and DMFS in a more pre-treated patient population [21]. The neoadjuvant melanoma landscape continues to evolve, offering new opportunities for personalized treatment based on individual tumor biology. While current evidence supports the use of neoadjuvant immunotherapy as a standard approach for resectable stage III/IV melanoma, addressing unresolved issues regarding biomarkers, personalized treatment strategies, and novel therapeutic modalities will be crucial to optimizing patient outcomes. In this context, the planned Multicentre Selective Lymphadenectomy Trial-3 (MSLT-3, NCT07049276) will be particularly important: by comparing selective index lymph node resection to standard therapeutic total lymph node dissection following NIT, the trial aims to determine whether less extensive surgery can preserve disease control while reducing morbidity and improving quality of life—findings that could substantially refine surgical management in the neoadjuvant setting.

## 4. Open Issues and Controversies

### 4.1. Adjuvant Treatment of Stage IIIA Melanoma

The AJCC 8th edition classification of stage III melanoma is complex and heterogeneous, ranging from stage IIID—the outcomes of which resemble those of metastatic melanoma—to stage IIIA, which is associated with a 5-year survival approaching 90% [22]. This staging system presents a paradox: stage IIIA melanoma has a substantially better prognosis than high-risk, sentinel lymph node (SLN)-negative primary melanoma (stage IIB–IIC). In addition, SLN positivity is defined irrespective of tumor size, tumor cell count, and the method of detection. Patients with stage IIIA disease were either excluded from pivotal adjuvant trials (e.g., CheckMate 238) or included only if the lymph node metastasis was >1 mm (e.g., KEYNOTE-054 and COMBI-AD). Subgroup analyses of these trials did not demonstrate an OS or RFS benefit. The absence of clear, compelling evidence that patients with stage IIIA melanoma benefit from adjuvant therapy has led to guidelines that emphasize clinician judgment and shared decision-making. The key question, therefore, is how to identify higher-risk patients who may warrant treatment. One important prognostic factor is the microanatomic location of the tumor deposit within the lymph node, as reflected by the Dewar classification [23] and, more recently, the sentinel node invasion level (SNIL) [24]. Other studies have focused on quantifying SLN tumor burden to identify subgroups with more favorable outcomes. In the study by Satzger et al., an SLN tumor burden < 1 mm was identified as a favorable prognostic factor in stage III melanoma [25]. Amaral et al. further showed that the presence of isolated melanoma cells in the SLN (<0.1 mm) is associated with outcomes comparable to stage IB melanoma, suggesting that these patients may not require systemic treatment (10-year RFS, 84% vs. 49%) [26]. In a multi-institutional registry study of 408 patients with stage IIIA melanoma with known SLN tumor burden, Moncrieff et al. reported a 5-year melanoma-specific survival (MSS) of 80.3% for patients with metastatic deposits ≥ 0.3 mm versus 94.1% for those with deposits < 0.3 mm (HR = 1.26, 95% CI 1.11–1.44; *p* < 0.0001) [27]. Notably, no significant survival differences were observed between patients with stage IIIA melanoma with deposits < 0.3 mm and their SLN-negative counterparts. For patients with tumor burdens of 0.3–0.9 mm, a recent retrospective analysis of 613 patients with stage IIIA melanoma found that greater Breslow thickness, a high mitotic rate, and nodal metastasis in the neck were associated with shorter RFS [28]. Another approach is to integrate clinical and pathologic variables into composite prognostic models. Stassen et al. developed a prediction model incorporating pathologic factors (ulceration, histologic subtype, Breslow thickness, sentinel node status, number of sentinel nodes removed, maximum diameter of the largest sentinel node metastasis, and Dewar classification) and clinical variables (sex, age, and primary tumor location) to predict 5-year RFS and MSS. Further development of this individualized prognostic approach is discussed in the section on translational data [29].

### 4.2. Overall Survival and Recurrence-Free Survival in Adjuvant Trials

Traditionally, in oncology, RFS has been used as a surrogate for OS. This is based on the principle that chemotherapy cannot cure established metastatic disease; therefore, preventing relapse offers the best chance of cure. However, this paradigm has shifted with the advent of IO, which, particularly in melanoma, is sufficiently effective to induce durable remissions (and likely cure) in a subset of patients with metastatic disease. This raises an important question: what proportion of patients who do not receive ADJ can be salvaged with the same treatment if and when they develop metastatic disease? The most influential study linking RFS with OS in melanoma is that of Suciu et al., who showed, in a meta-analysis of high-risk stage II–III melanoma treated with interferon or ICIs, that RFS is a valid surrogate for OS [30]. However, to date, ipilimumab is the only ADJ that has demonstrated an OS benefit versus placebo, in an era when the most effective therapies for relapse were not yet available [31]. Despite clear improvements in RFS and DMFS, none of the adjuvant trials with anti-PD-1 therapy or dabrafenib plus trametinib have demonstrated an OS benefit. Thus, the magnitude of benefit for the most clinically meaningful endpoint for melanoma patients who are candidates for ADJ remains incompletely defined. This is particularly relevant when considering absolute benefit: in stage III disease, the absolute improvement in RFS is approximately 17–20% [32], whereas it is approximately 12% in stage IIB–IIC disease based on KEYNOTE-716 [33]. A more direct way to evaluate net benefit is the number needed to treat (NNT) and its relationship to the number needed to harm (NNH), which reflects toxicity-related harm. In stage IIB–IIC disease, the margin between benefit and harm is particularly narrow (NNT 7.8 and NNH 6.8) [34]. One approach to addressing these uncertainties is the crossover design adopted by some trials. For example, KEYNOTE-054 allowed patients who relapsed after being initially randomized to placebo to cross over to pembrolizumab. More trials are expected to incorporate similar designs in the future.

### 4.3. Retreatment at Relapse After Adjuvant Therapy

As adjuvant treatment expands to earlier disease stages, an increasing proportion of patients who present with metastatic relapse in routine practice will have previously received ICIs or TT. Optimal management in this setting remains an area of active investigation. Because most landmark trials were conducted in systemic treatment-naïve populations, robust prospective data are limited. In the most recent report of KEYNOTE-054, the 5-year RFS2 rate was 68.2% in the pembrolizumab group; at first recurrence, anti-PD-1-based therapy, anti-CTLA-4 therapy, and TT were administered to 27.2%, 18.4%, and 14.7% of patients, respectively [35]. Four years after the first recurrence, the proportion of patients alive and free of progression and second recurrence was 21.8% and 24.3% in the pembrolizumab and placebo groups, respectively, suggesting that initiating treatment at metastatic relapse may partially mitigate prognosis in a subset of patients. Nevertheless, among patients previously treated with pembrolizumab who relapsed >6 months after completing therapy and were rechallenged with pembrolizumab, the response rate was only 15%, with a median PFS of 4.1 months. Systemic therapy may retain activity in this context; however, response rates vary by drug class and by whether recurrence occurs during (on-treatment) or after (off-treatment) ADJ. In a retrospective study, Owen et al. described outcomes among patients who relapsed during (ON) or after (OFF) adjuvant anti-PD-1 therapy. Ipilimumab-based regimens and BRAF/MEK inhibitors appeared most active at relapse, with response rates of 26% (10/38) and 82% (27/33), respectively. These findings are broadly consistent with data in advanced melanoma, in which response rates after anti-PD-1 failure are approximately 30% with subsequent anti-CTLA-4-based immunotherapy [36]. However, response rates with ipilimumab monotherapy versus ipilimumab plus anti-PD-1 were similar—an unexpected observation, given retrospective data in advanced melanoma suggesting higher activity with combination therapy than with single-agent ipilimumab after anti-PD-1 failure [37]. Among patients who recurred ON adjuvant anti-PD-1 therapy, none (0/6) responded to anti-PD-1 rechallenge, 24% (8/33) responded to ipilimumab (alone or in combination with anti-PD-1), and 78% (18/23) responded to TT. Among those who recurred OFF adjuvant anti-PD-1 therapy, 40% (2/5) responded to anti-PD-1 monotherapy, 40% (2/5) to ipilimumab-based therapy, and 90% (9/10) to TT. Other real-world studies have confirmed poor outcomes among patients who progress during ADJ and the limited effectiveness of anti-PD-1 retreatment in the setting of early progression [38]. Although the optimal interval remains uncertain, a minimum washout of 3–6 months has been proposed before considering retreatment with the same agent, as emphasized by the SITC Immunotherapy Resistance Taskforce [39]. These studies also highlight a substantial risk of early systemic recurrence after an initial resectable locoregional relapse. Taylor et al. recently explored the effectiveness of a “second adjuvant” strategy with BRAF/MEK inhibitors in patients who develop locoregional or oligometastatic recurrence despite adjuvant PD-1-based immunotherapy. This approach was associated with improved RFS (median RFS 30.4 vs. 4.0 months), albeit with immature OS data and at the cost of substantial toxicity [40]. In conclusion, before considering systemic retreatment, local, regional, and distant relapse should be distinguished, as they have different clinical implications. Wide local excision remains essential for local control and adjuvant systemic therapy should not be viewed as a substitute for adequate surgery. In patients with isolated local or locoregional recurrence, complete resection with appropriate margins should be considered whenever feasible, followed by multidisciplinary reassessment of recurrence risk and systemic treatment options according to updated stage, BRAF status, prior adjuvant therapy, disease-free interval, and patient-related factors.

## 5. Translational Data

### 5.1. Biomarkers in Adjuvant Therapy

**Figure 2:**
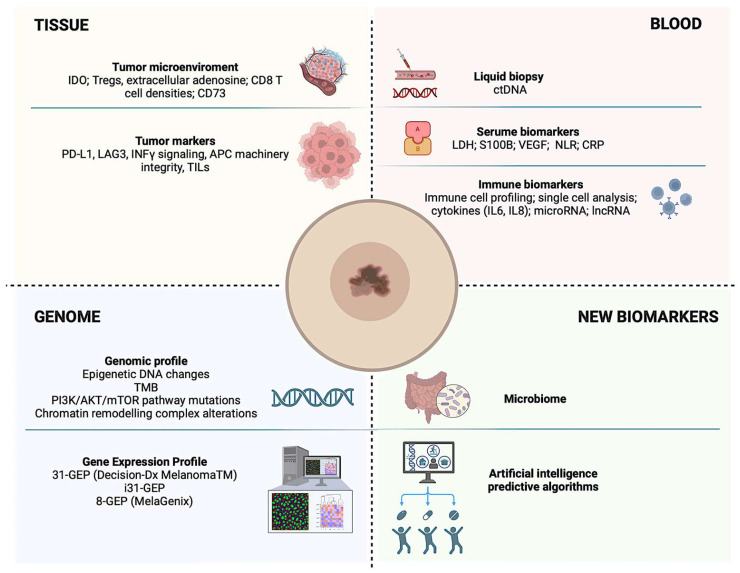
Candidate biomarkers in adjuvant and neoadjuvant melanoma. Blood-based markers, ctDNA, tumor microenvironment features, immune gene-expression signatures, TMB, PD-L1 expression, microbiome composition, and gene-expression profiling may refine risk stratification, but most remain investigational and require prospective validation before routine clinical use.

Despite substantial progress in melanoma treatment, a considerable proportion of patients treated with ICIs do not benefit or experience disease progression after an initial response. Biomarker development is therefore crucial to improve the balance between the number needed to treat and the number needed to harm, by identifying patients most likely to benefit from ADJ or NIT and sparing others unnecessary toxicity. Although several candidate biomarkers have been investigated (Figure 2), no robust, clinically actionable predictor of benefit is currently established for routine use in the adjuvant or neoadjuvant melanoma setting. Blood-based markers, including lactate dehydrogenase, S100B, C-reactive protein, neutrophil-to-lymphocyte ratio, and other inflammatory parameters, are feasible and minimally invasive, but their role in the adjuvant setting remains mainly prognostic and not yet clinically actionable [41]. Circulating tumor DNA (ctDNA) has emerged as a promising biomarker across multiple tumor types. In melanoma, recent studies have shown that ctDNA detection after surgery predicts relapse in patients with stage II–III disease [42] and that detectable preoperative ctDNA is prognostic in this population. Collectively, these data suggest that ctDNA can identify patients at higher risk of early relapse and poorer survival. This concept is being prospectively evaluated in the DETECTION study (NCT04901988), which uses ctDNA to identify high-risk patients for treatment rather than treating all comers. Tumor-based biomarkers have also been explored, including PD-L1 expression, tumor mutational burden, CD8-positive T-cell infiltration, IFN-gamma-related gene-expression signatures, and other features of the tumor microenvironment, including the analysis of the immunocompetent cell subpopulations [43,44]. Exploratory analyses from adjuvant and neoadjuvant trials suggest that immune-enriched tumors may be associated with improved outcomes after immune checkpoint blockade [45,46,47]. However, these markers have not yet demonstrated sufficient reproducibility, predictive accuracy, or clinical utility to support treatment selection in routine practice [48,49,50,51,52,53,54]. Overall, biomarker research in resected melanoma remains highly promising but still immature. Future studies should prioritize prospective validation, standardized assays, clinically meaningful cutoffs, and treatment-selection designs capable of demonstrating whether biomarker-driven strategies can reduce overtreatment, identify patients at high risk of relapse, and improve long-term outcomes.

### 5.2. Improve Prognostication of Stage II Melanoma: Focus on Gene Expression Profile (GEP)

Because of their high incidence, early-stage thin melanomas account for a substantial number of melanoma-related deaths each year. Following the demonstrated RFS and DMFS benefits in stage IIB–IIC melanoma, major regulatory agencies have approved anti-PD1 for this indication. However, the proportion of patients who benefit from treatment is lower than in stage III disease, and many patients who are already cured by surgery alone are nevertheless exposed to potentially long-term toxicities. For example, immune-related effects involving the pituitary, thyroid, pancreas, and adrenal glands may lead to chronic endocrinopathies that can persist lifelong. Identifying patients most likely to benefit from ADJ—while sparing those with low recurrence risk—therefore remains a key unmet need. Moreover, a subset of patients at genuinely high risk may be underestimated by conventional anatomic staging alone. Gene expression profile (GEP) assays are molecular tests that measure the expression of a panel of validated genes and provide prognostic information, including the risk of recurrence, metastasis, and melanoma-specific death [55]. This approach has already proven useful in other malignancies, including uveal melanoma [56]. The most widely studied commercially available assay is the 31-gene expression profile test, DecisionDx-Melanoma. This assay evaluates the expression of 28 discriminating genes and 3 control genes and classifies patients into risk groups, generally reported as low risk, intermediate risk, or high risk. Several retrospective and prospective observational studies have shown an association between high-risk GEP results and worse clinical outcomes, including lower RFS, DMFS, and melanoma-specific survival (MSS). In particular, patients classified as high risk have consistently shown higher rates of recurrence and distant metastasis compared with those classified as low risk. These associations have also been reported in patients with stage I–II disease, suggesting that GEP may identify biologically aggressive tumors among patients who would otherwise be considered relatively low risk according to AJCC staging alone [57,58]. Evidence supporting the 31-GEP assay includes validation studies and meta-analyses showing that GEP class can provide prognostic information independent of conventional clinicopathologic factors. For example, high-risk GEP classification has been associated with significantly worse RFS and DMFS compared with low-risk classification. Moreover, integrated models combining the 31-GEP result with clinicopathologic features, such as patient age, Breslow thickness, ulceration, mitotic rate, tumor-infiltrating lymphocytes, histologic subtype, and anatomic location, appear to improve individualized risk prediction compared with either clinicopathologic variables or GEP alone. This integrated approach may be particularly relevant for patients with stage I–II melanoma, in whom the decision to intensify surveillance or consider adjuvant therapy is often clinically challenging. Another commercially available assay, MelaGenix, has also been investigated as a prognostic tool in primary cutaneous melanoma. This test uses a gene-expression signature to stratify patients according to metastatic risk and has been reported to improve prognostication when combined with conventional staging. However, compared with the 31-GEP assay, the amount of published clinical validation is more limited, and broader external validation in contemporary cohorts remains necessary. Amaral et al. demonstrated the clinical utility of the Merlin Assay (CP-GEP), a molecular test that evaluates GEP in patients with clinical stage I/II melanoma who did not undergo SLNB [59]. One of the key advantages of the Merlin Assay is its simpler read-out, using a streamlined 8-gene GEP panel. This not only makes the assay potentially more accessible for routine clinical practice but also provides clinicians with clear, actionable results that could guide treatment decisions. From a clinical perspective, GEP assays may have several potential applications. First, they may support more individualized surveillance strategies by identifying patients at higher risk of recurrence who could benefit from closer clinical and radiologic follow-up. Second, they may help identify patients who are candidates for clinical trials evaluating adjuvant systemic therapy or biomarker-driven escalation strategies. Third, in selected low-risk patients, GEP results might contribute to discussions about de-escalation of follow-up intensity or the likelihood of benefit from additional interventions. Some studies have also explored the use of GEP assays to guide sentinel lymph node biopsy decisions, particularly in patients with tumors in whom the probability of sentinel node positivity is borderline. However, this application remains controversial and should not replace established clinicopathologic criteria or guideline-based recommendations. Despite these promising data, several limitations must be emphasized. Most evidence supporting GEP assays is prognostic rather than predictive. Therefore, while these tests may help estimate recurrence risk, they do not currently demonstrate that a patient with a high-risk GEP result will derive greater benefit from adjuvant immunotherapy or targeted therapy. In addition, many validation studies were conducted before the widespread use of modern adjuvant systemic therapy, and the performance of these assays in patients treated with contemporary anti-PD-1 or BRAF/MEK inhibitor therapy requires further evaluation. Another important limitation is the need for prospective studies showing clinical utility—that is, evidence that GEP-guided decisions actually improve patient outcomes, reduce unnecessary treatment, or optimize surveillance in a cost-effective manner. Accordingly, GEP assays should currently be interpreted as complementary tools that may refine prognostic assessment, rather than as standalone determinants of treatment selection. Their results should be integrated with AJCC stage, Breslow thickness, ulceration, mitotic rate, sentinel lymph node status, patient comorbidities, treatment preferences, and multidisciplinary clinical judgment. In the future, GEP may become more clinically actionable if incorporated into prospective trials together with other biomarkers such as circulating tumor DNA, tumor mutational burden, immune gene-expression signatures, and tumor microenvironment features.

## 6. Conclusions

The therapeutic landscape of resected melanoma has evolved substantially. Adjuvant anti-PD-1 therapy and BRAF/MEK inhibition have improved outcomes in stage III and resected stage IV disease, and anti-PD-1 therapy has extended the benefit to stage IIB-IIC melanoma. Neoadjuvant immunotherapy is now strongly supported by randomized data for selected patients with clinically detectable, resectable stage III/IV melanoma, but its optimal use requires multidisciplinary expertise and further refinement of response-adapted strategies. At the same time, these advances highlight the limitations of treatment decisions based mainly on anatomic stage. The absolute benefit of adjuvant treatment varies considerably across subgroups, particularly in stage IIIA and stage IIB-IIC disease, where the risk of overtreatment and long-term toxicity is clinically relevant. Emerging approaches, including personalized mRNA neoantigen vaccines, novel checkpoint inhibitor combinations, oncolytic viruses, and intralesional immunocytokines, are promising but should remain cautiously framed until mature randomized evidence is available. Future progress will depend on integrating clinical stage, pathological features, molecular risk, ctDNA, tumor microenvironment features, gene-expression profiling, and pathological response to neoadjuvant therapy into practical treatment algorithms. Such a personalized model may allow clinicians to intensify therapy for patients at high risk of relapse while safely de-escalating or avoiding treatment in patients with a low probability of benefit.

## Data Availability

No new data were created or analyzed in this study. Data sharing is not applicable to this article.
